# Structure and functions of Aggregation-Induced Emission-Photosensitizers in anticancer and antimicrobial theranostics

**DOI:** 10.3389/fchem.2022.984268

**Published:** 2022-08-30

**Authors:** Heidi Abrahamse, Michael R. Hamblin, Sajan George

**Affiliations:** ^1^ Laser Research Centre, University of Johannesburg, Doornfontein, South Africa; ^2^ School of Bio Sciences and Technology, Vellore Institute of Technology, Vellore, TN, India

**Keywords:** aggregation-induced emission, laser, light, nanoparticles, photosensitizers, theranostics

## Abstract

Photosensitizers with Aggregation-Induced Emission (AIE) can allow the efficient light-mediated generation of Reactive Oxygen Species (ROS) based on their complex molecular structure, while interacting with living cells. They achieve better tissue targeting and allow penetration of different wavelengths of Ultraviolet-Visible-Infrared irradiation. Not surprisingly, they are useful for fluorescence image-guided Photodynamic Therapy (PDT) against cancers of diverse origin. AIE-photosensitizers can also function as broad spectrum antimicrobials, capable of destroying the outer wall of microbes such as bacteria or fungi without the issues of drug resistance, and can also bind to viruses and deactivate them. Often, they exhibit poor solubility and cellular toxicity, which compromise their theranostic efficacy. This could be circumvented by using suitable nanomaterials for improved biological compatibility and cellular targeting. Such dual-function AIE-photosensitizers nanoparticles show unparalleled precision for image-guided detection of tumors as well as generation of ROS for targeted PDT in living systems, even while using low power visible light. In short, the development of AIE-photosensitizer nanoparticles could be a better solution for light-mediated destruction of unwanted eukaryotic cells and selective elimination of prokaryotic pathogens, although, there is a dearth of pre-clinical and clinical data in the literature.

## Introduction

Natural products such as curcumin, hypericin, hypocrellin, riboflavin and many synthetic compounds such as, tetrapyrroles, phenothiaziniums, rose bengal and squaraine have been widely explored for their photodynamic activity in living tissues (Abrahamse and Hamblin, 2016). Further, photosensitizers such as Foscan, Fimaporfin, Hemoporfin, Redaporfin, Talaporfin sodium, Verteporfin, Photolon, Photosens and Tookad have been investigated in preclinical and clinical trials (Hamblin, 2020). These photosensitizers are characterized by excitation to a long-lived triplet state and transfer energy upon light illumination, thereby converting oxygen molecules to highly reactive singlet oxygen and free radicals. However, photosensitizers with Aggregation-Induced Emission (AIE) are able to replace conventional photosensitizer nanomaterials in theranostics due to their ease of synthesis and biological compatibility, enhanced permeability and retention effect, excellent fluorescence properties as well as their capacity for efficient photoacoustic imaging in living cells. They do undergo tissue aggregation with less autofluorescence making them very effective for Photodynamic Therapy (PDT) (Wang et al., 2021). Additionally, the ability of AIE-photosensitizers to ‘light-up' in response to an external stimuli or changes in their microenvironment makes them suitable for theranostic applications (Wang et al., 2018a).

Nanomaterials such as Au/Ag nanoparticles, mesoporous/magnetic/polymeric nanoparticles, carbon nanotubes/graphene, as well as quantum dots have been widely used as theranostic platforms (Kang et al., 2020). Several of these organic and inorganic nanomaterials coupled with AIE-photosensitizers may allow direct delivery into cellular organelles for studies of cellular processes and facilitate image-guided therapies. These nanoparticles have an increased surface area for drug delivery and they are responsive to near infrared wavelengths, just as AIE-photosensitizers. Currently, many of these AIE-photosensitizer nanoparticles have *absorption maxima* below 500 nm and *emission spectra* below 700 nm, although, it is desirable to have *absorption spectra* with a narrow band gap and *emission maxima* around 800 nm for the best biological effects (Korneev et al., 2019). These AIE-photosensitizers are quite useful for cancer therapy and can overcome resistance to antibiotics in microbes. This review explores the mechanisms of AIE-photosensitizers on cancer and also the photophysical properties against infectious agents.

## History of photosensitizers

The *first* generation photosensitizers examined for clinical use were porphyrin-based compounds such as hematoporphyrin derivative marketed as Photofrin^®^, which faced significant drawbacks in terms of skin photosensitization as well as a low molar absorption coefficient (Table 1). They were soon replaced by the *second* generation photosensitizers with significant modifications to the porphyrin core structure. The *third* generation non-porphyrin photosensitizers have the advantages of activation by longer wavelengths, shorter photosensitization periods and higher yield of singlet oxygen, although, they still have the tendency to aggregate in a polar environment and a lack of specificity (Zhang et al., 2018a). These drawbacks are attributed to the flat aromatic structures of photosensitizers resulting in the π-π stacking in the aggregated state, which is referred as Aggregation-Induced Quenching (AIQ). On the contrary, the molecular structures of AIE-photosensitizers take advantage of the aggregation of photosensitizers in concentrated solutions as well as in solid state to perform better than any of the conventional systems (Luo et al., 2001).

**TABLE 1:** Commonly used photosensitizers and their mode of action.

Generation	Mode of action	References
First		
Hematoporphyrin	Absorbs light forming an excited triplet state and converting oxygen to ROS and free radicals	Kou et al. (2017)
Photofrin^®^	Photofrin^®^ induces apoptosis of cancer cells through mitochondrial caspase-3 pathway	Baran, (2018)
Second		
Phthalocyanine	Localization in cellular organelles and occlusion of tumor-associated vasculature	Roguin et al. (2019)
Aminolevulinic acid (5-ALA)	Metabolic conversion of aminolevulinic acid to protoporphyrin IX generates singlet oxygen	Li et al. (2021a)
Third		
Chlorin e6 NP	Production of singlet oxygen causing stress in endoplasmic reticulum and mitochondrial damage	Wawrzyńska et al. (2010)
BODIPY-type	Higher yield of triplet excited state with halogen atoms loaded in the organic chromophore	Mitra et al. (2016)
Next		
T-TPETS nanodots	Targeted-TPETS nanodots cause necrosis or apoptosis at high- and low-doses, respectively	Gao et al. (2019a)
Upconversion Nanoparticles	Upconversion Nanoparticles are activated by near infrared wavelengths for deeper tissue penetration	Wang et al. (2021)

AIE, Aggregation-Induced Emission; EDTA, Ethylene Diamine Tetra acetic acid; NP, Nanoparticles; ROS, Reactive Oxygen Species.

## Mechanism of Aggregation-Induced Emission-photosensitization

PDT requires an optimal concentration of oxygen, duration and wavelength of light along with appropriate structural and photophysical properties and tissue distribution of the photosensitizer molecules (Silva et al., 2015). AIE-photosensitizer molecules have a propeller-like configuration, which allows the spatial orientation of conjugated units connected by single bonds. Their rotation will be accelerated in dilute solutions, especially during light irradiation. These rotations in dilute solution consume most of their absorbed energy while emitting weak or no luminescence. However, when these molecules are aggregated, the rotations of the conjugated units are inhibited due to the enhanced intermolecular interactions. This leads to the blockade of non-radiative decay and the energy released from the excited state of the molecule is seen as fluorescence or as phosphorescence (radiative decay) (Ni et al., 2021). In fact, the propeller like configuration of AIE-photosensitizers (referred to as rotors) is responsible for the intramolecular motion caused by the absorption of energy in dilute solutions. They emit only minimal or no light in dilute solutions while having large “Stokes Shift” upon aggregation. Their rotors possess intense non-radiative dissipation pathways in dilute solutions, but in the aggregated state they show radiative decay to emit fluorescence, and Intersystem Crossing (ISC) to the triplet state and photodynamic activity (Liu et al., 2021). In short, there will be restriction of their molecular motion during aggregation in concentrated or solid states, referred as Restricted-Intramolecular-Rotation (RIR) or Restricted-Intramolecular-Vibration (RIV), which will activate the radiative decay channel for emitting strong fluorescence (Wang et al., 2015).

**FIGURE 1:**
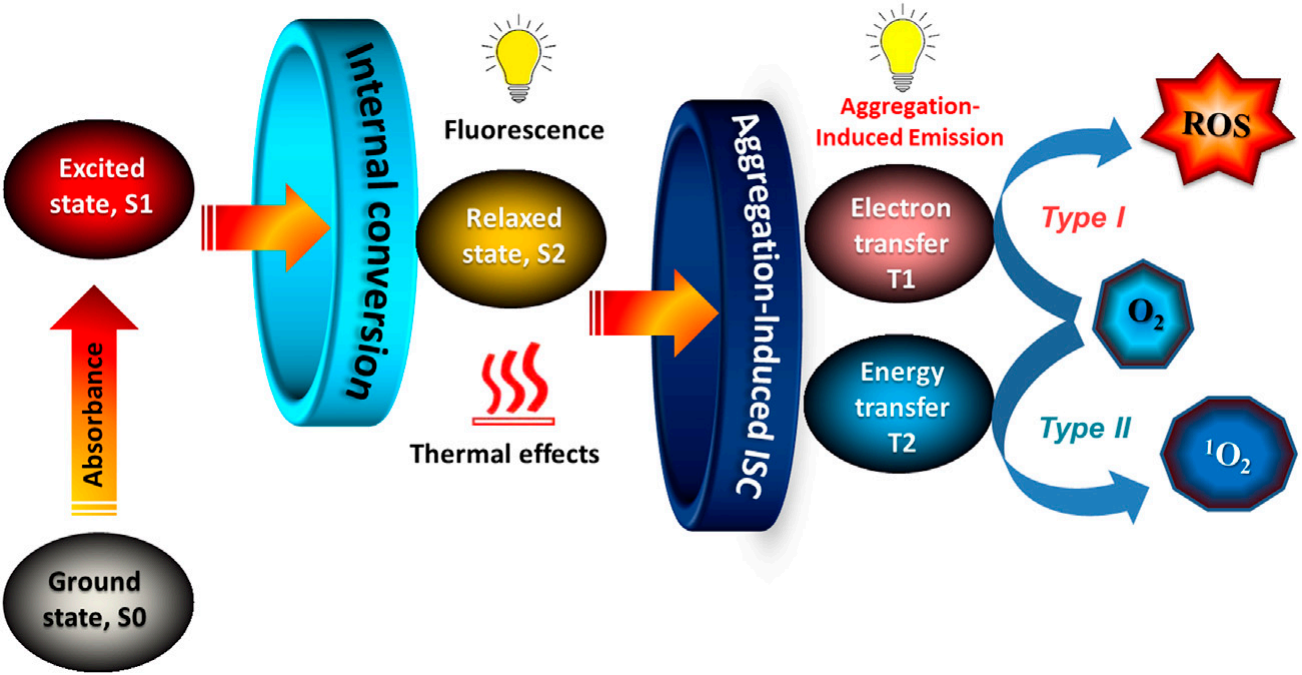
Mechanism of AIE-photosensitization: AIE-photosensitizers at the ground state (S0) absorbs energy to become excited Singlet states (S1, S2), which then undergoes Intersystem Crossing (ISC) to the Triplet states for the transfer of electrons (T1) or energy (T2). Production of free radicals and singlet oxygen can be increased in T1 or T2 reactions by accelerating ISC from S1 to S2. Thus, it is ideal to have lower energy gap between S1 and S2 and a large spin-orbit coupling. Further, design of the AIE-photosensitizer molecules with a D-structure should allow aggregation-induced ISC. Unlike any other fluorescent dyes, AIE-photosensitizers can overcome Aggregation-Induced Quenching (AIQ) in their condensed state and are most suitable for theranostics. *Abbreviations: O*_*2*_ *, Oxygen;* ^*1*^ *O*_*2,*_ *Singlet oxygen; ROS, Reactive Oxygen Species.*

The functionality of an AIE-photosensitizer commences with the absorption of a photon by its ground state (S0) to the electronic excitation states (S1 and S2). Subsequently, they can undergo ISC from their singlet (S1) state to the excited triplet state (T1) leading to the formation of free radicals by *type I* (electron transfer) or *type II* (energy transfer) resulting in the production of Reactive Oxygen Species (ROS) or reactive singlet oxygen (^1^O_2_) from ground state triplet oxygen (Figure 1). Since the advent of AIE-photosensitizers, several advances and modifications have been reported to increase the yield of fluorescence and free radicals including singlet oxygen. An increase in the production of oxide free radicals, especially singlet oxygen can be achieved by increasing the ISC from the lowest excited state (S1) to the lowest triplet state (T1). To accomplish this, the singlet high energy gap (Δ*E*_ST_), which is the energy gap between singlet and triplet states and a large spin-orbit coupling, should be minimal. One of the ways to reduce Δ*E*_ST_ is by designing AIE-photosensitizers with a D-structure or with a higher ISC quantum yield (Hu et al., 2018). Further, ∆*E*_ST_ can be minimized for an efficient singlet oxygen production by inserting conjugated moieties as linkers or spacers (*π*) between the *donor* and *acceptor* moieties. Accordingly, the AIE-photosensitizer core structure consists of *neutral*, *donor* and *acceptor*. And, they are divided into 1) Donor-AIE (neutral)-acceptor and 2) AIE (donor)-acceptor.

Studies have shown that inserting conjugated moieties as linker(s) between the *donor* and *acceptor* moieties may lengthen the space between the Highest-energy Occupied Molecular Orbital (HOMO) and the Lowest-energy Unoccupied Molecular Orbital (LUMO), thereby minimizing the ∆*E*_ST_ and increasing singlet oxygen yield of the photosensitizers (Xu et al., 2015; Ni et al., 2021). An increased separation between the HUMO and LUMO reduces the electronic repulsion and the resultant lower Δ*E*_ST_ may improve the efficiency of ROS production. Thus, an effective separation of HOMO and LUMO results in less conjugation between *acceptor* and *donor* moieties leading to short wavelength absorption and emission. However, the combined *acceptor* moieties may results in a “red-shift” of absorption and emission due to the strong electron withdrawing ability of the two *acceptors*. The combined *acceptor* and *donor* moieties connected by a double bond give better conjugation resulting in a “red-shift” of absorption and emission (Liu et al., 2019a; Alam et al., 2020).

## Cellular effects of Aggregation-Induced Emission-photosensitizers

**FIGURE 2:**
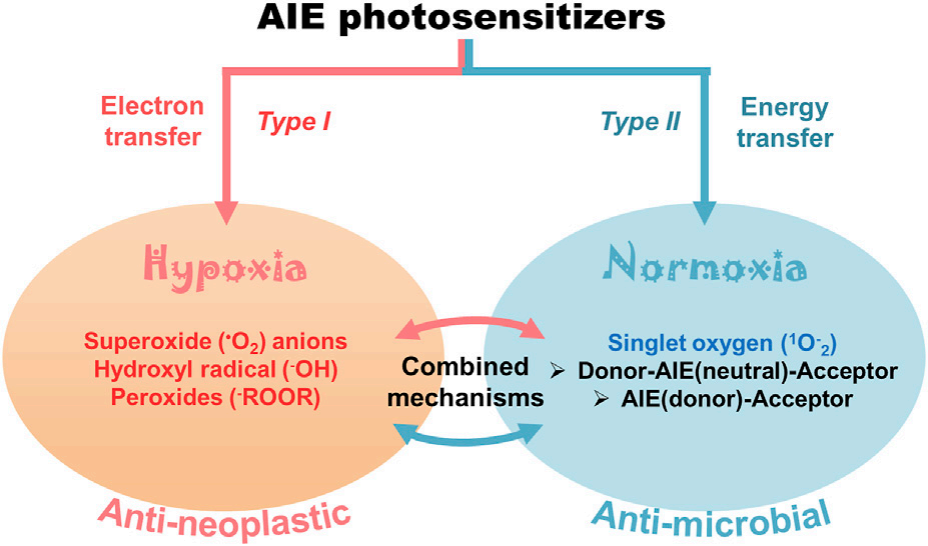
Cellular effects of AIE-photosensitizers: The AIE-photosensitizers are classified as *type I* and *II* according to their process of synthesis of ROS. Transfer of electrons at low oxygen concentration creates oxide free radicals (*type I* photosensitization), while transfer of energy to the molecular oxygen generates singlet oxygen (*type II* photosensitization). Often, electron transfer may happen from AIE-photosensitizer to oxygen in *type II* reactions forming superoxide anions. The *type II* photosensitization is subdivided into 1) Donor-AIE (neutral)-Acceptor 2) AIE (donor)-Acceptor, and 3) combined electron and energy transfer mechanisms. While the *type I* photosensitization has anti-neoplastic effects due to the robust oxidation of biomolecules, the latter is mostly anti-microbial. Paradoxically, *type II reaction* with electron transfer mechanisms has been widely used in anticancer theranostics.

The activity of AIE-photosensitizers relies mainly on their retention in living tissues and their photophysical mechanisms. Accordingly, they are classified into three categories 1) electron transfer; 2) energy transfer; 3) combined mechanisms (Figure 2). *Type I* reaction involves transfer of the electrons at low oxygen concentrations (hypoxia) leading to the formation of superoxide radical anions. Usually, this is followed by the formation of hydroxyl radicals and peroxides in tumors *in vivo* as well as in spheroids in 3-dimensional cell cultures. Here, the electron transfer may happen in either directions, but usually, the excited photosensitizer will act as an oxidant. The *type I* photochemical mechanism can also occur where the electrons transfer from the AIE-photosensitizers to oxygen for the formation of superoxide and other free radicals. On the contrary, *type II* involves energy transfer from the triplet state of AIE-photosensitizer to the ground state of molecular oxygen (also a triplet) under normal oxygen concentrations (normoxia). The net result is the formation of reactive singlet oxygen, which is detrimental to both cancers as well as microbes. However, this hypothesis needs to be rigorously tested with various AIE-photosensitizers for proper validation.

PDT can be much more effective either by direct killing of tumor cells or by closing blood vessels immediately surrounding the tumor mass leading to hypoxia and necrosis. In general, AIE-photosensitizers are effective in metastatic tumors depending on the abundant supply of oxygen and blood for their rapid growth and spread (Wang et al., 2021). However, AIE-photosensitizers with *type I* ROS formation are adapted to the hypoxic microenviroment around the tumor tissues and, therefore be a better therapeutic choice. Rapidly growing tumors are metabolically active requiring more oxygen and nutrients, which is achieved by the process of neovascularization. For this reason, most of the photosensitizers synthesized and used for PDT are *type II* nowadays. These molecules require sufficient oxygen tension in the cellular microenvironment for their optimal activity. However, rapidly growing tumors as well as deep seated tumors fails to meet the demand of oxygen and nutrients due to their impedance in angiogenesis. Frequent exposure to PDT also creates an oxygen deprived condition leading to the ineffectiveness of *type II* reactions. Thus, there is an imminent need to identify and develop AIE-photosensitizers, which is much tolerant and effective in hypoxic environment with *type I* reaction for ROS formation (Zhou et al., 2016; Ni et al., 2021).

## Theranostic applications of Aggregation-Induced Emission-photosensitizers

AIE-photosensitizers should have controllable excitation for absorbing many wavelengths of light, which will increase their activity on living cells, especially tumors and microbes. They need also to be excited using near infrared irradiation rendering them active against deep seated tumors. Often, the rational design of AIE-photosensitizers can result in long wavelength excitation peaks. The intramolecular charge transfer due to the strong electron D-A interaction as well as extended π-conjugation also results in the absorption and emission of longer wavelengths (Gao et al., 2019b; Wu et al., 2019). AIE-photosensitizers, TTPy and MeTTPy were synthesized using this approach and demonstrated excellent tumor targeting and ROS generation capabilities. They possess better absorption and emission at longer wavelengths, far red and near infrared regions. TTPy shows better penetration, less cytotoxicity as well as good anti-bacterial effects (Wang et al., 2018b; Wang et al., 2021).

AIE-photosensitizers do not undergo AIQ due to their hydrophobic and intrinsically rigid planar structures in contrast to more traditional fluorophores (Liu et al., 2021). Quite often, they are accumulated in the cell membrane phospholipids, phosphatidylethanolamine intercalated with phosphatidylserine. Upon activation by light (photons) these AIE-photosensitizers with either electron transfer or energy transfer properties (classified as *type I* or *type II*) can produce a range of ROS. In fact, the appropriate *donor* and *acceptor* in the molecular skeleton of AIE is responsible for lowering the Δ*E*_ST_ by π-π stacking and a higher ISC, which results in higher ROS production (Wang et al., 2021). AIE-photosensitizers with *type II* reactivity are constructed as D-A or D-π-A to keep the Δ*E*_ST_ as low as possible and can either be configured as Donor-AIE (neutral)-acceptor or as AIE (donor)-acceptor. There are instances, when the AIE-photosensitizers may generate both free radicals and singlet oxygen by both *type I* and *II* reactions. These can be referred as “combined mechanisms” involving transfer of electrons as well as energy between the *donor* and *acceptor* molecules. Below we summarize the AIE-photosensitizers commonly used for cancer and microbial theranostics based on the information gleaned from available literature (Tables 2–5). Unfortunately, many of these findings from various sources fail to disclose the “structure-activity relationship” of AIE-photosensitizers, which make our classification incomplete.

**TABLE 2:** AIE-photosensitizers for anticancer theranostics.

AIE-photosensitizer	Structure and activity	References
Berberine chloride (AIE-photosensitizer from natural source)	Anti-inflammatory and antioxidant activity. It can selectively stain and eliminate Gram positive bacteria and metastatic cancers	Lee et al. (2019)
TPAN, TPAPy	TPAPyPF6 can target mitochondria and produce singlet oxygen in lipid environment	Liu et al. (2019b)
TPE-4EP+	Anchored on the mitochondria and constant irradiation will lead to apoptosis of cancer	Zhang et al. (2019a)
TPE-MEM	AIE-photosensitizer with compatibility, water solubility and specificity on cell membranes	Zhang et al. (2019b)
TPPM, TTPM	Triphenylamine (***electron donor***) and methyl pyridinium (***electron acceptor***)	Zhuang et al. (2019)
IVP-02, 22, 42, 62	They help to monitor the viability of cancers using mitochondrial-nucleolar fluorescence	Zhang et al. (2020a)
TTVPHA, TTVPHE	Cationic AIE localizes in mitochondria due to electrostatic attraction and lipophilic effect	Zhou et al. (2020)
TPBPy	TPBPy has a donor-π-acceptor structure with TPB (***electron donor***) and pyridinium (***electron acceptor***) for two-photon imaging	He et al. (2021a)
MP-TPEDCH	TPEDCH (***electron donor***) and lysosomal targeting fraction 4-(2-chloroethyl) morpholine (***electron acceptor***)	Huang et al. (2021)
TBD-R	Tetraphenylethene (***electron donor***), benzothiadiazole (***electron acceptor***), phenyl (π) and dicyanovinyl (***electron acceptor***) cause cancer pyroptosis	Wu et al. (2021a)
PyTPE with paclitaxel	A reduction-sensitive polymeric prodrug, PMPT is added to AIE photosensitizer, TPA-BDTP (TB) to form TB@PMPT.	Yi et al. (2021)
MeOTPPM	An electron-rich anion photosensitizer with plasma membrane permeability and AIE.	Zhao et al. (2021)

The bold italics indicate the functional role of each molecule.

**TABLE 3:** AIE-photosensitizer nanoparticles for anti-cancer theranostics.

AIE-nanoparticles	Structure and activity	References
DPBA-TPE	Dimethoxybenzene and arylamines (***electron donor***), tetraphenylethene (***neutral***) and cyano groups (***electron acceptor***). DPBA-TPE gains entry through the cell surface folate receptors	Feng et al. (2015a)
PTPEAQ-NP-HER2	Tetraphenylethene (***electron donor***) and anthraquinone (***electron acceptor***) with a conjugate polymer, Anti-HER2 for cell targeting	Wu et al. (2016)
TPETCAQ nanoparticles	The HOMO-LUMO distribution of TPEDC1 and TPEDC2 (***electron donor***) allows electron transfer through TPE (***neutral***) to TPETCAQ (***electron acceptor***) with emission at near infrared region	Wu et al. (2017a)
TPE-pHP-Cbl	Tetraphenylethene is coupled with the p-hydroxy phenacyl-chlorambucil conjugate and nanoparticle. It can produce singlet oxygen under white light and release anti-cancer drug for theranostics	Parthiban et al. (2018)
TB1-RGD dots	AIE-dots decorated with the c-RGD peptide to form high quantum yield TB1-RGD dots with absorption peak at the near infrared region for tumor targeting	Sheng et al. (2018)
DTE-TPECM	Tetraphenylethene (***electron donor***) and malononitrile (***electron acceptor***) forms smart nanoparticles switching from closed to open ring structure for imaging and therapeutic effects	Qi et al. (2018)
TPE-Br with DSPE-PEG2000	TPE-Br nanoparticles coated with DSPE-PEG2000. They have higher singlet oxygen and fluorescence	Yang et al. (2018)
MeO-TPE-indolium with polydopamine as nanoparticles	D-π-A conjugated structure with MTi (***electron donor***), tetraphenylethene (***neutral***) and Indolium (***electron acceptor***). MTi targets mitochondria	Chen et al. (2019)
T-TPETS nanodots	Targeted-TPETS nanodots cause necrosis or apoptosis at high- and low-doses, respectively	Gao et al. (2019a)
CeOx, graphite-C_3_N_4_, metformin, and upconversion nanoparticles	These upconversion nanoparticles are combined with CeOx, graphite-C_3_N_4_ and the anticancer drug (metformin) to overcome cellular hypoxia	Jiang et al. (2019)
Au^I^-NHC with PPh_3_ ligand	Gold(I) N-heterocyclic carbene (Au^I^-NHC) complex. Au^I^-NHC with PPh_3_ ligand binds to the target and inhibit antioxidant enzyme (thioredoxin reductase)	Zhang et al. (2020b)
TPA with BODIPY	This has Donor-Acceptor-Donor structure with Triphenylamine (***electron donor***) connected to BODIPY (***electron acceptor***) by thiopene	Deng et al. (2021)
	This BODIPY-based compound is transformed into nanoparticles with AIE by twisted structure of TPA.	
TPA-diCN	Triphenylamine (***electron donor***) and 6,7-dicyanoquinoxaline (***electron acceptor***). Core made of TPA-diCN and shell with pluronic F127 to give large Stokes shift and near infrared emission	Mao et al. (2021)
TBD-Br on DNAzyme forming nanoparticle	TBD-Br is grafted to phosphorothiolated DNAzyme backbone. It disrupts lysosomal structure causing escape of Zn-ligated DNAzyme nanoparticles	Shi et al. (2021)
AIEPS5 with PEG2000	AIEPS1 methoxy-substituted TPE (***electron donor***), fumaronitrile (***auxiliary acceptor***) and dicyanovinyl (***electron acceptor***). They are dispersible in water for targeted delivery with anti-Her-2 nanobody in tumor xenograft models	Wu et al. (2021b)
Au585@AIEPS nanodots	TPATCN encapsulated in DSPE-mPEG polymer. Au585@AIEPS nanodots produce higher ROS that exhibits better fluorescence and photosensitivity	Yaraki et al. (2021)
TBP with Au(I)	TBP-Au consists of TBP (***electron donor***) and anticancer Au(I) (***electron acceptor***) nanoparticles	Zou et al. (2021)

The bold italics indicate the functional role of each molecule.

**TABLE 4:** AIE-photosensitizers for antimicrobial theranostics.

AIE-photosensitizer	Structure and activity	References
AIE with vancomycin	AIE-2Van produce ROS causing damage to the cell walls of Gram positive bacteria	Feng et al. (2015b)
TPE-Bac	Tetraphenylethene (***electron donor***) and pyridinium (***electron acceptor***). Two positively charged amines and two long alkyl chains of TPE-Bac intercalates with bacterial membrane	Zhao et al. (2015)
Triphenylethylene-Naphthalimide Triazole (TriPE-NT)	TriPE is useful for imaging while NT is antibacterial. Together they have broad spectrum bactericidal activity	Li et al. (2018)
PyTPE-CRP conjugate (Caspase-1 Responsive Peptide)	Tetraphenylethene (***electron donor***) and pyridinium (***electron acceptor***). Cellular co-localization is achieved by CRP peptide and the mitochondria targeting by pyridinium. PyTPE aggregates creating singlet oxygen	Qi et al. (2019)
TPE-Cy fluorescence varies with cellular pH	*E. coli* shows a strong blue fluorescence in co-culture with cells and macrophages	Gu et al. (2019)
AIE-photosensitizer conjugated phages	Function as both AIE and bacteriophage for real-time monitoring and bacterial targeting	He et al. (2020)
AIE-photosensitizer TPACN with D-Alanine	Diphenylamine **(*electron donor*)** and dicyano vinyl **(*electron acceptor*).** TPACN is a metabolic probe for *in vivo* tracking and eradicating bacteria from biofilms	Mao et al. (2020)
AIE-photosensitizer 4TPA-BQ (organic salt)	Triphenylamine (***electron donor***) and benzoquinoline (***electron acceptor***). 4TPA-BQ shows small Δ*E*_ST_ for higher singlet oxygen	Li et al. (2020)
Receptor-targeting AIE-photosensitizer, CE-TPA	Cephalothin is conjugated to cationic D–A type as antimicrobial AIE-photosensitizer	Wang et al. (2022a)

The bold italics indicate the functional role of each molecule.

**TABLE 5:** AIE-photosensitizer nanoparticles for antimicrobial theranostics.

AIE-nanoparticles	Structure and activity	References
DTF-FFP nanoparticles are anti-microbial with *type II* reaction	DTF (***energy donor***) and FFP (***energy acceptor***). It is used for imaging of endogenous bacteria	Wu et al. (2020)
TPA-2PE, TPA-PCN, TPA-2PCN with polystyrene maleic anhydride	Nanoparticles binds to the surface of bacteria by hydrogen bonds	Yuan et al. (2021)
AIE-photosensitizer is loaded on nanofibrous membrane, TTVB	TTVB has a donor-π-acceptor (D-π-A) structure with triphenylamine (***electron donor***), vinylthiophene (π) and 3-ethylbenzo [d]thiazol3-ium (***electron acceptor***)	Li et al. (2021b)

The bold italics indicate the functional role of each molecule.

## Aggregation-Induced Emission-photosensitizers in anticancer theranostics

**FIGURE 3:**
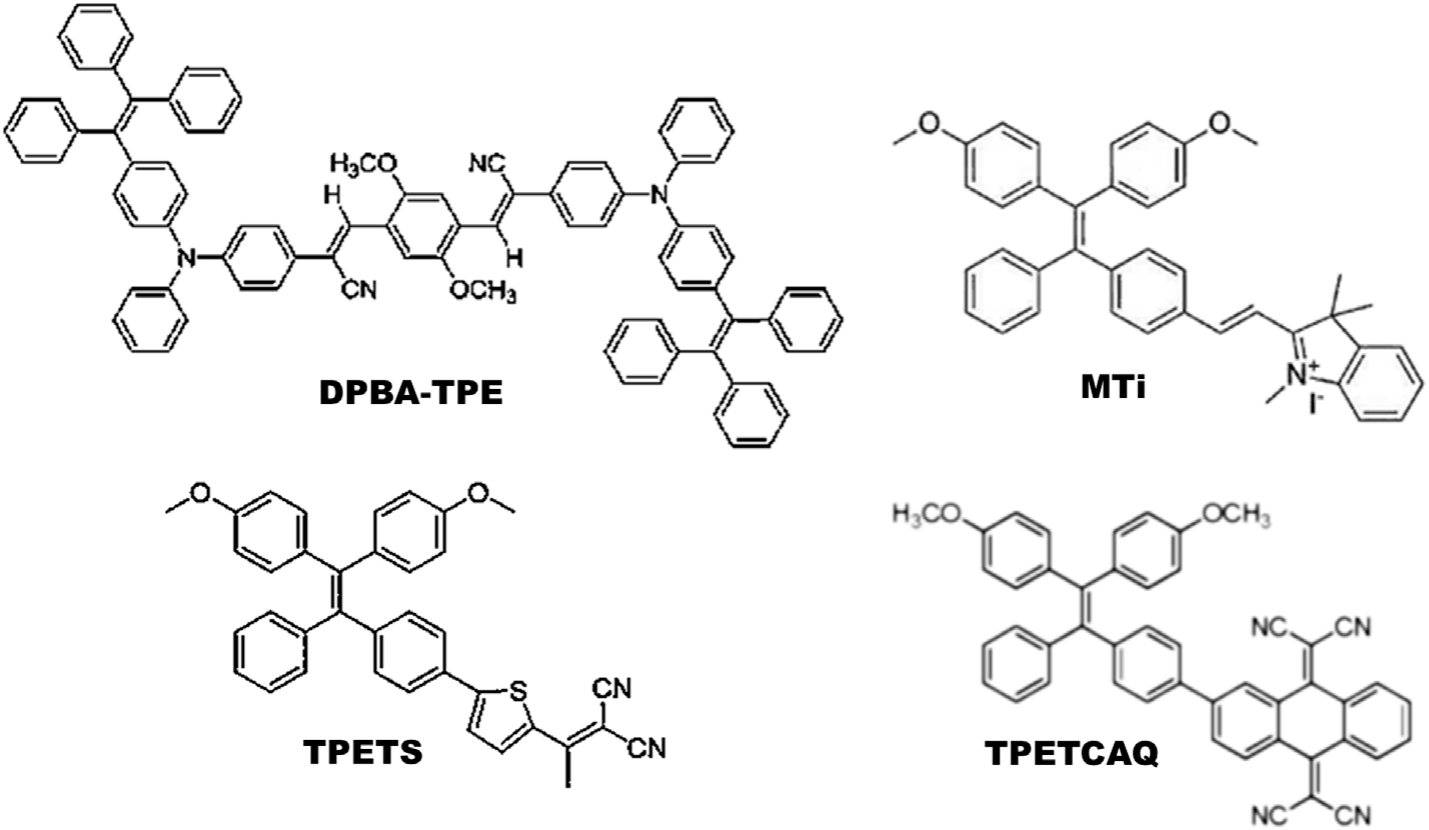
Illustrations for Donor-AIE(neutral)-Acceptor photosensitizers: In general, these are constructed on a tetraphenylethene (TPE) cytoskeleton. Alternatively, tetraphenylamine (TPA) can act as an electron donor and/or core of the AIE-photosensitizer. Source: DPBA-TPE (Feng et al., 2015a), MTi (Chen et al., 2019), TPETS (Gao et al., 2019a), TPETCAQ (Wu et al., 2017a). Please refer Tables 2–5 for details.

**FIGURE 4:**
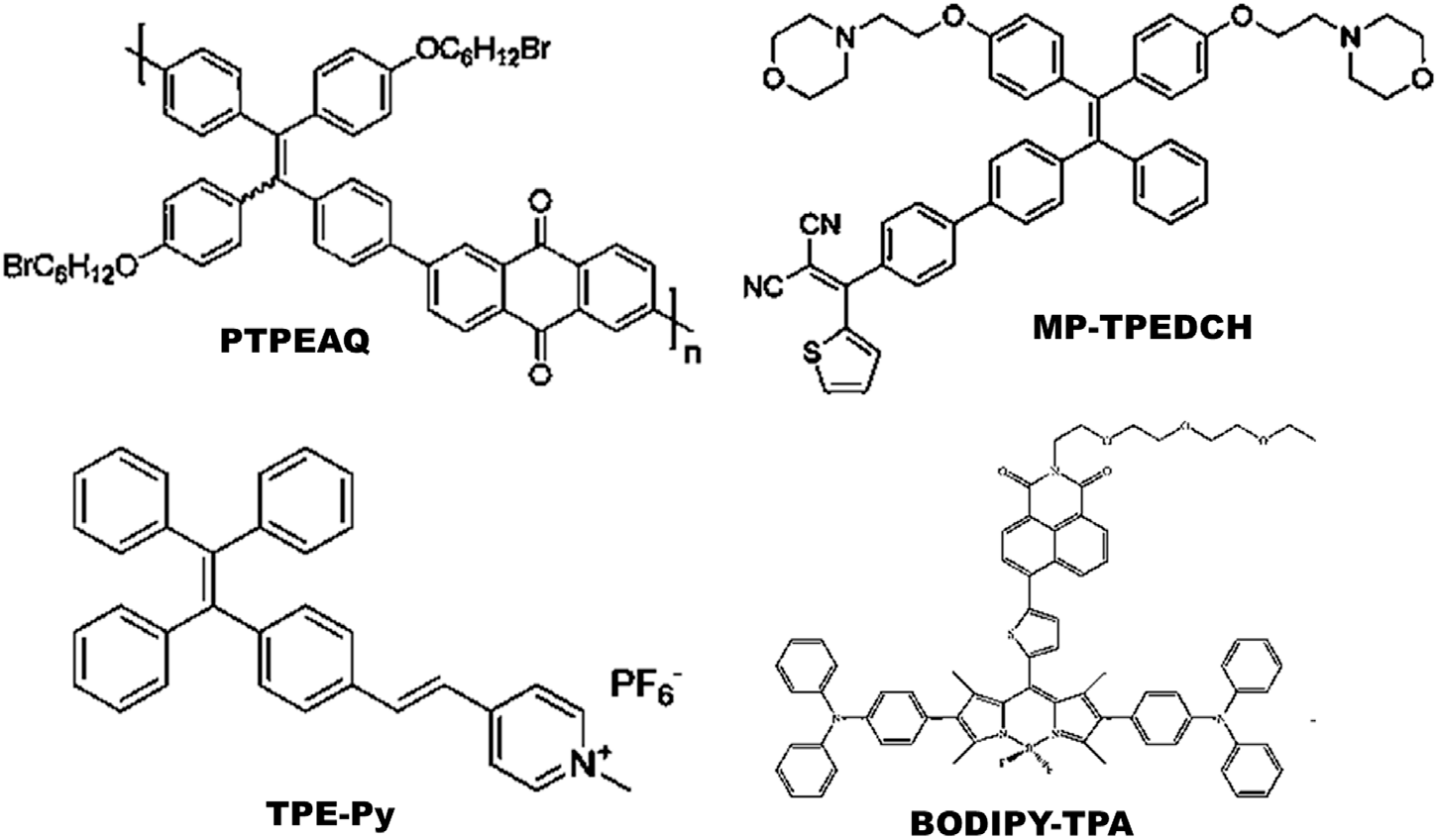
Illustrations for AIE(donor)-Acceptor photosensitizers: These are constructed on tetraphenylethene (TPE) cytoskeleton with a D-A or D-π-A structure. Addition of spacer (*π*) between *donor* (D) and *acceptor* (A) may decrease the singlet energy gap (Δ*E*_ST_). Source: PTPEAQ (Wu et al., 2016), MP-TPEDCH (Huang et al., 2021), TPE-Py (Zhuang et al., 2019), BODIPY-TPA (Deng et al., 2021). Please refer Tables 2–5 for details.

Most commonly synthesized and widely used AIE-photosensitizers are classified as Donor-AIE (neutral)-acceptor or AIE (donor)-acceptor, constructed on a tetraphenylethene (TPE), triphenylamine (TPA) and rarely on a triarylamine (TAA) to form a D-A or D-π-A structure (Figures 3, 4). These structures are efficient in the production of ROS and singlet oxygen upon interaction with living cells by *type I* and *type II* reactions respectively. Most of the newly synthesized AIE-photosensitizers exhibit *type II* reactions, although, they are more suitable on rapidly growing or metastatic cancers receiving abundant oxygen and nutrients.

## Aggregation-Induced Emission-photosensitizer nanoparticles for anticancer theranostics

Metallic nanoparticles containing heavy metals such as gold, silver, platinum or titanium have the ability to provide hydrophobicity in the cellular environment, which is necessary for the entry of photosensitizers and overcoming biological barriers (Crous and Abrahamse, 2020). The new generation nanoparticles such as fullerenes, titanium dioxide or quantum dots may improve the cellular delivery and photochemical internalization of genetically encoded protein photosensitizers (Tynga and Abrahamse, 2018). The AIE-photosensitizer nanoparticles possess effective biodistribution properties with strong optical absorption and scattering properties. Noble-metal nanoparticles can carry out localized surface plasmon resonance to increase their light absorption, while interacting with cellular molecules (He et al., 2021b). However, there is lack of evidence on the nature of ROS produced by various AIE-photosensitizers nanoparticles while interacting with the living cells *in vitro* and *in vivo*.

## Aggregation-Induced Emission-photosensitizers in antimicrobial theranostics

AIE-photosensitizers can be constructed for microbial detection with precision from various environmental samples. Chen at al. (2014) used a fluorescent sensor array with five AIE probes to detect and differentiate eight different bacteria from water by flow cytometry and analysis. AIE-photosensitizers have ‘turn-on’ characteristics after aggregation and allow fluorescence imaging of infectious agents (Liu et al., 2021). They adhere more to the thick layer of peptidoglycan of Gram-positive bacteria than to the thin phospholipid membrane of Gram-negative bacteria (Bai et al., 2021). Yet, they may bind to Gram-negative bacteria by electrostatic action and destroy them. AIE-photosensitizers could also bind with the cell wall of drug-resistant fungi and their spores (Chen et al., 2022).

## Aggregation-Induced Emission-photosensitizer nanoparticles for antimicrobial theranostics

AIE-phtotosensitizers were used to generate bacteriophage as AIE–PAP bioconjugate with capabilities for real-time tracking by fluorescence imaging and killing of antibiotic-sensitive or multi-drug-resistant bacteria (He et al., 2020). AIE-photosensitizer nanoparticles with an overall positive charge can act as broad spectrum antimicrobials activated by near infrared light to produce singlet oxygen and heat (Wang et al., 2022b). Glucose polymer-modified gold nanoparticles were incubated with diverse types of bacteria to be taken up through the ATP-binding cassette (ABC) transporter pathway before laser irradiation to achieve a three-fold increase in the microbicidal activity (Yang et al., 2022). Detailed studies are required to establish the sensitivity/specificity of nanoparticles on environmental and food pathogens.

## Biological effects of Aggregation-Induced Emission-photosensitizer nanoparticles

Nano-drug delivery systems include dendrimers, liposomes, micelles, carbon nanotubes as well as various nanoparticles viz. gold, silver, zinc, magnetic, virus etc. All these make use of the gap junctions and breaches in the capillaries to selectively accumulate in tumors with an Enhanced Permeability and Retention (EPR) effect. Among these, gold nanoparticles have the highest cellular uptake as well as an increased production of singlet oxygen due to the surface plasmon resonance for enhancing the effects of PDT (Kruger and Abrahamse, 2018). Conventional photosensitizers are hydrophobic requiring any of these nano-drug delivery systems to gain entry into neoplasms by crossing gap junctions of cells. However, AIE-photosensitizers incorporated in organic nanoparticles show EPR, strong luminescence and good photostability without AIQ property. Moreover, nanomaterials as aggregates of AIE-photosensitizers (AIE-dots) have unique potential in theranostics (He et al., 2021b).

**FIGURE 5:**
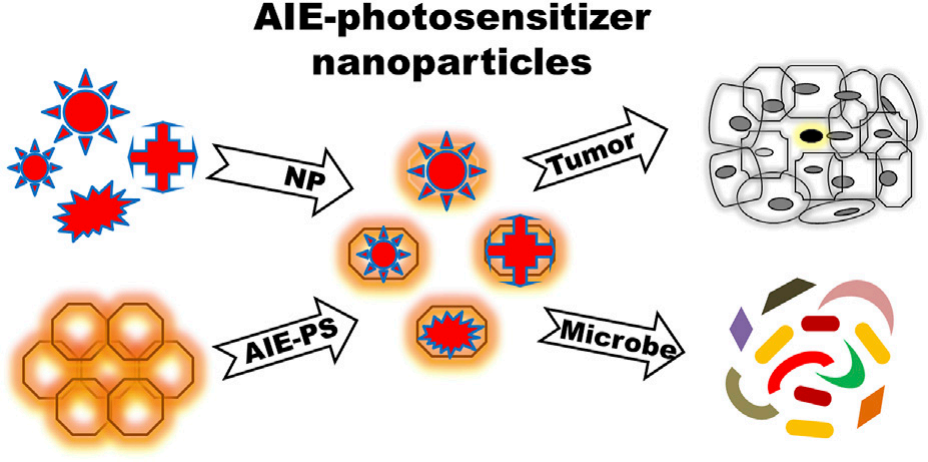
Mode of action of AIE-photosensitizer nanoparticles: A selected number of biocompatible nanoparticles can be incorporated into AIE-photosensitizers for an efficient drug delivery with desirable biological effects. AIE-photosensitizer nanoparticles with *emission maxima* in the far-red to near-infrared have high penetration depth into tumors, which is useful for bio-imaging as well as targeted therapy in PDT. Further, they are able to detect specifically and eliminate pathogens from various environment and food sources. *Abbreviations: AIE-PS, Aggregation-Induced Emission-Photosensitizer; NP, Nanoparticles.*

Nanoparticles can exert selective activity against tumors, providing increased bioavailability of photosensitizers to rapidly dividing cells with minimal side-effects. However, nanoparticles such as carbon black, carbon nanotubes, copper and zinc are not only toxic to hepatocytes but also sensitive to tissues, such as alveoli and neurons (Yao et al., 2019). They may cause endoplasmic reticulum stress, multiple organelle dysfunction and also affect mitochondrial dynamics (fusion-fission) resulting in cytochrome c-dependent apoptosis or mitophagy. Nanotoxicity refers to the capacity of the nanomaterials to alter cellular morphology and function, thereby reducing metabolic activity and cellular viability. Nanotoxicity can be a reason for either blockade or induction of autophagy, and can cause lysosomal rupture leading to oxidative stress and inflammation (Liu and Tang, 2020). On the contrary, AIE-photosensitizers encapsulated in organic nanomaterials or synthesized as AIE-dots can perform target-specific destruction of cancers and microbes with least toxicity (Figure 5).

A suitable absorption/emission spectrum is essential for the use of AIE-photosensitizers in image-guided therapy. In a study, Yu et al. (2017) has shown that a mitochondria-anchored AIE-photosensitizer generated a high yield of singlet oxygen under white light leading to the apoptosis of cancer, while enabling its visualization under fluorescence “turn-on” mode. Similar functions were also shown by TPE-IQ-2O, which not only distinguishes cancers from healthy cells but also generates ROS upon irradiation with white light (Gui et al., 2017). The biotinylated AIE-photosensitizer, TPE-TETRAD, demonstrates differential staining of cancers from healthy cells and stains mitochondria with emission at the far red region under two/three-photon fluorescence microscopy (Nicol et al., 2017).

The dynamics of ROS production in living cells changes when using AIE-photosensitizers loaded into nanoparticles. Wu et al. (2017a, 2017b) reported an efficient yield of singlet oxygen by a newly synthesized AIE-photosensitizer (please refer Table 3) incorporated in nanoparticles capable of absorption in the ultraviolet-visible region and with stable emission in far red to near infrared region (700–1,000 nm). These AIE-photosensitizers demonstrated low dark toxicity, good photostability as well as good biocompatibility in BALB/c mice, which make them useful for image-guided anticancer PDT. In fact, the recent additions to the list of AIE-photosensitizers with emission in the far infrared region (1,000–1,500 nm) show superior tissue penetration and higher fluorescence emission suitable for photoacoustic dual-mode imaging as well as a tunable photothermal effect (Liu et al., 2020a). Interestingly, TBL dots with F127 emit chemiluminescence under infrared light (Liu et al., 2020b).

AIE-photosensitizers excited by near infrared irradiations can be modified by connecting tetraphenylethene with atypical AIE (donor)-acceptor molecules for the production of Upconversion Nanoparticles (UCNPs). They efficiently aggregate and generate ROS under near infrared irradiation in solutions with higher fractions of water (Dai et al., 2020). These UCNPs are efficient in converting near infrared to shorter wavelengths of visible light for the activation of photosensitizers. It is proposed that the combination of UCNPs and photosensitizers will improve the penetration depth and therapeutic efficacy of PDT (Zhang et al., 2007). Another attempt has been made using AIE-photosensitizers with amphiphilic polymers encapsulating hydrophobic UCNPs. This UCNP@AIE-cRGD formulation could maintain fluorescence intensity under near infrared illumination for a long period and induce apoptosis of tumors residing at a depth up to 6 mm in xenograft rodent models (Jin et al., 2019). The effect of near infrared irradiation on the mitochondrial energy transfer mechanism of living cells is detailed by George et al. (2022).

## Biomedical applications of Aggregation-Induced Emission-photosensitizer nanoparticles

Organic nanoparticle t-BPITBT-TPE aggregates encapsulated with DSPE-mPEG micelles shows good biocompatibility and biodistribution in the zebra fish embryo. The *in vitro* grown cancer cell lines tagged with t-BPITBT-TPE in polymeric nanoparticles efficiently track them for their growth and metastasis in zebra fish larvae and in Balb/c mice (Lin et al., 2017). These fluorescent photosensitizer nanoparticles with AIE properties when tested in a transparent zebra fish larvae with inducible liver hyperplasia gave an understanding of the biodistribution of nanoparticles helpful for the screening of various AIE-photosensitizer nanoparticle *in vivo* (Manghnani, et al., 2018). Furthermore, this study shows a correlation between the uptake of nanoparticles, dosage of light and the duration of trigger activation.

Gu et al. (2018) found that cancer theranostics can be boosted by restricting intra-particle microenvironment using corannulene-incorporated AIE-photosensitizer nanoparticles. Corannulene limited the intramolecular rotation thereby facilitating the fluorescence pathway and ISC of the AIE-photosensitizers in tumor xenograft models. Similarly, aptamer-AIE organic nanomaterials precisely targets cancer cells using their aptamer-cholesteryl and PEG-lipid moieties for biosensing as well as imaging (Zhang et al., 2018b). Organic nanodots targeted to integrin α_ν_β_3_ and bearing AIE-photosensitizers activated by red light are also developed for image-guided PDT and theranostics. These targeted PETS nanodots demonstrates a high yield of singlet oxygen causing dose- and time-dependent apoptosis resulting in the ablation of hepatocellular carcinoma (Gao et al., 2019a).

Nanocarriers are useful for tumor targeting of photosensitizers and reduce the toxicity to normal healthy cells. In one particular study, a pH-responsive AIE-photosensitizer without a nanocarrier was tested in a tumor-bearing mouse model for efficacy and safety. These AIE nano-photosensitizers are self-assembled from amphiphilic AIE-photosensitizers, and avoided the protonation and deprotonation of the carboxyl groups resulting in a higher ROS yield and good treatment efficiency under white light (Cheng et al., 2020). These changes in the redox potential are quite sensitive, and often an increase in the level of oxidative stress hinders cancer (George and Abrahamse, 2020). In another study, three photosensitizer nanostructures made up of 2,3-bis(4′-(diphenylamino)-[1,1′-biphenyl]-4-yl) fumaronitrile (BDBF) are encapsulated in Pluronic F-127 nanoparticles, which shows better ability to generate ROS with AIE characteristics for imaging and tumor regression. The encapsulation in Pluronic F-127 nanoparticles improves the ultrastructure of BDBF, which could self-assemble as nanorods or spherical structures for therapeutic benefits (Han et al., 2020).

Redox-sensitive AIE nanoparticles are developed as micelles of poly (ethylene glycol) (PEG) and cholesterol (CE) conjugated disulfide-containing polyamido amines are found to be useful for fluorescence imaging. These TPE-MI encapsulated micelles show a red shift and an increased fluorescence emission according to the concentration of cellular glutathione, thus mimicking the redox potential of the cells (Cheng et al., 2014). AIE dye-loaded polymer nanoparticle formulations are developed using Pluronic F127 and PEGylated phospholipid with deep-red emission for siRNA delivery, and could be used as nanovectors for gene silencing of mutant *K-ras* in pancreatic cancer (Hu et al., 2014). Imaging of cells is also accomplished using AIE nanoparticles with nucleic acid induced peptide co-assembly, which emitted fluorescence in response to an increasing concentration of nucleic acids. This allows real time monitoring of drug release from peptide-based nanocarriers *in vivo* and *in vitro* (Li et al., 2021c). AIE-photosensitizer nanoparticles are also useful tools for tracking the metastasis of cancers as well as differentiating neurons with high penetration and retention in neuronal cells (Jang et al., 2021).

## Conclusion

AIE-photosensitizers are useful for detecting and eliminating tumors and microbes in the tissue environment. After PDT there will be an additional restriction of nutrients and oxygen, especially due to the photochemical consumption of oxygen during the continuous PDT process. *Type I* AIE-photosensitizers are active even in hypoxic environments rendering them suitable for use in poorly-perfused solid tumors. This property of *type I* AIE-photosensitizers makes them useful for *in vitro* PDT assays using 3-dimensional spheroids. On the contrary, other AIE-photosensitizers are *type II*, and their optimal activity may require oxygen supply to living cells. Thus, the latter may be more effective as microbicidal agents.

Aggregates of AIE-photosensitizers with restricted intermolecular motion reduce the loss of energy by radiative decay, while, relaxation of the excited state results in fluorescence and ISC. Aggregation also results in the increased energy transfer from the excited singlet to triplet states thereby reducing the energy gap (Δ*E*_ST_) and resulting in the higher production of ROS (Yang et al., 2016). Another strategy to improve the yield of ROS at the triplet state is by modifying the *donor* and acceptor (D-A) structures and perhaps, adding a spacer (D-π-A) to stabilize the incoming electrons towards generating free radial anions (Zhou et al., 2016). These modifications may also extend the excitation/emission of AIE-photosensitizers to wavelengths in the infrared region, making them ideal choice for theranostics. Furthermore, characteristics of the two/three-photon excitation can be achieved by extending conjugation length of the π-system, which will increase their cross-sectional distance. This will make AIE-photosensitizers suitable for two/three-photon excitation for 3-dimensional imaging in greater depth and detail with least amount of photobleaching and autofluorescence.

AIE-photosensitizers and AIE-dots can be tailored with controllable excitation wavelengths, which may increase their penetration depth in tissues affected with tumors or infections. ROS generation is found to be higher in stimulus-responsive AIE-photosensitizers, which also have improved water solubility and can carry out photothermal effects for sustained photosensitization (Wang et al., 2021). Some AIE-active nanomaterials such as graphene, quantum dots etc. have been proven useful for drug delivery, optoelectronics, as well as theranostics. However, more efficient methods for the qualitative and quantitative detection of ROS in cells, and the use of two/three-photon excitation PDT should be investigated for future development of AIE-photosensitizers and AIE-dots in theranostics.

## Fundings

This research is supported by the South African Research Chairs Initiative (SARCHI) of the Department of Science and Technology and National Research Foundation of South Africa (Grant No. 98337). SG is supported by the Vellore Institute of Technology (VIT), Vellore, TN, India. MH was supported by US NIH Grants R01AI050875 and R21AI121700.
